# SFTSV Prevalence in Ticks and Livestock in an SFTSV-Endemic Area in Central China

**DOI:** 10.3390/pathogens14090944

**Published:** 2025-09-18

**Authors:** Hui-Ya Lu, Guan-Du Wu, Meng Peng, Li-Bang Wu, Yi-Ming Luo, Bin Xia, Dan Xiong, Xiang-Rong Qin, Fang Guo, Xue-Jie Yu

**Affiliations:** 1 State Key Laboratory of Virology, School of Public Health, Wuhan University, Wuhan 430071, China; lu_huiya@163.com (H.-Y.L.); m18934685562@163.com (G.-D.W.); lem0917@whu.edu.cn (Y.-M.L.); zerifa@163.com (B.X.); 2 Suizhou City Disease Prevention and Control Center, Suizhou 441300, China; peng.m@foxmail.com (M.P.); szjkwlb@163.com (L.-B.W.); kirazxxl@163.com (D.X.); 3 Department of Clinical Laboratory, The Second Qilu Hospital of Shandong University, Jinan 250000, China

**Keywords:** ticks, goats, SFTSV, China

## Abstract

Severe fever with thrombocytopenia syndrome virus (SFTSV) is an emerging tick-borne bunyavirus that causes a severe viral hemorrhagic fever (SFTS), with a very high case mortality rate, expanding epidemic areas, and increasing incidence. Due to the lack of an effective drug or vaccine for SFTS, reducing the incidence and mortality of SFTS primarily relies on decreasing the density of ticks and the number of their host animals. However, which tick species and vertebrate animal serve as the major reservoir and animal host of SFTSV are not clearly understood. In May of 2023 and June of 2024, we collected 2437 ticks from domesticated animals and grassland in Suizhou City, a prefecture of Hubei Province in central China. A total of 195 domesticated animal blood samples were collected, including 152 goats, 26 cattle, and 17 dogs. Ticks were grouped for RNA extraction according to their life stages and feeding status. RNA from each animal’s blood and each group of ticks was extracted with an RNA extraction kit and tested for SFTSV with RT-PCR. Ticks were classified according to morphology, and representative ticks of each stage were confirmed with PCR amplification and DNA sequencing of the mitochondrial 16S RNA gene. Among the collected ticks, the majority were from goats (72.7%, 1772/2437), and *Haemaphysalis longicornis* was predominant, accounting for 99.47% (2425/2437), and other tick species were very rare, with 0.45% (11/2437) *Rhipicephalus microplus*, and 0.04% (1/2437) *H. flava* and *Ixodes sinensis*, respectively. We found SFTSV RNA in *H. longicornis* ticks with a minimum infection rate of 0.17% (4/2424) and in one goat (0.66%,1/152). In summary, we demonstrated that the *H. longicornis* tick is positive for SFTSV and that the goat is the major host of *Haemaphysalis longicornis* in Suizhou, central China. Our study suggests that controlling ticks on goats may play an important role in preventing SFTSV infection in China.

## 1. Introduction

Ticks are obligate blood-feeding arthropods that can transmit pathogens to humans and animals through bites. In 2009, a severe viral hemorrhagic fever known as severe fever with thrombocytopenia syndrome (SFTS) was discovered in central China, which is caused by a tick-borne bunyavirus named after the disease, as sever fever with thrombocytopenia virus (SFTSV) [1]. The primary clinical symptoms of SFTS are fever, fatigue, muscle pain, thrombocytopenia, and leukopenia, with a case mortality rate ranging from 16 to 30% [1,2,3,4]. Currently, there are no effective drugs or vaccines for SFTS, and the primary approach is symptomatic treatment. SFTSV is a negative-sense, single-stranded RNA virus belonging to the genus *Bandavirus* in the *Phenuiviridae* family [1,5]. SFTSV is primary transmitted through tick bites, but can also be transmitted to humans from patients or infected animals such as cats, dogs, and mink [6,7,8,9] through mucous membranes or aerosol [10,11,12]. From 2010 to 2021, a total of 18,968 SFTS cases were reported in 27 provinces of China, with the number of cases increasing yearly and endemic areas continuing to expand [13]. Most of the SFTS cases (99.3%) have been concentrated in the hilly and mountainous forest areas of central China (Henan, Shandong, Anhui, Hubei, and Jiangsu) [14], and it has also been reported in South Korea, Japan, and Southeast Asian countries [15,16,17,18,19,20]. The hard tick *Haemaphysalis longicornis* has been demonstrated as a vector and reservoir of SFTSV, and it is the most abundant tick species collected in endemic areas in East China and South Korea [21,22,23,24]. The central region of China was the first to discover SFTSV and is also the area most severely affected by SFTSV [1,13]. However, we are not clear about the tick species and the major vector of SFTSV in central China. The aim of this study was to investigate tick species and the prevalence of SFTSV in ticks and domesticated animals in Suizhou City, Hubei Province, in central China. Suizhou City is an SFTS endemic area [25], with 277 and 248 cases in 2023 and 2024, respectively, according to surveillance data from the Chinese Center for Disease Control and Prevention.

## 2. Materials and Methods

### 2.1. Sample Collection Sites

**Figure 1:**
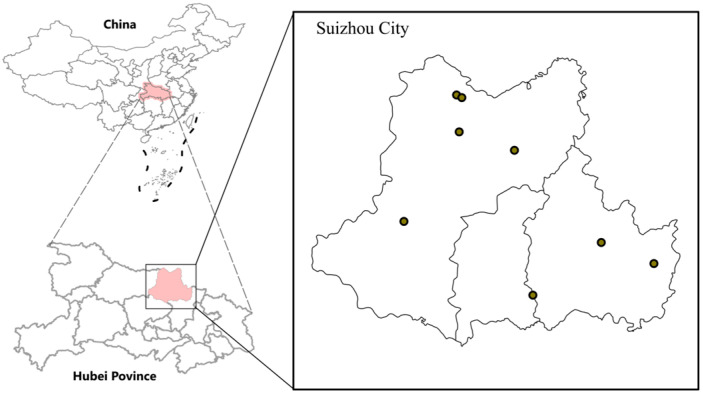
Sampling sites in Suizhou City, Hubei Province.

Suizhou City (31°19′ N to 32°26′ N, 112°43′ E to 113°46′ E) is a prefecture-level city located in northern Hubei Province in central China (Figure 1). The landscape of Suizhou City is dominated by low mountains, hills, and alluvial plains. Suizhou has a monsoon climate, with distinct seasons, abundant rainfall, and an average annual precipitation of 865 to 1070 mm across most regions. The region is rich in vegetation, with over 50% forest coverage, including subtropical evergreen broadleaf forests and deciduous broadleaf forests.

### 2.2. Animal Blood and Tick Collection

In May of 2023 and June of 2024, we collected questing ticks on grasslands and feeding ticks from goats, dogs, and cattle in rural areas in eight towns in Suizhou City, Hubei Province (Figure 1). The towns included Gaocheng, Wanhedo, Yindian, Wushan, Xiaolin, Hedian, Xihe, and rural areas under the jurisdiction of Beijiao Street. Sampling was conducted within a 5 km radius around residences of reported SFTS cases.

Questing ticks were collected by flagging grasses near farms and feeding ticks were collected from goats, cattle, and dogs with tweezers. According to the number of parasitic ticks on each host, 20 to 40 parasitic ticks were collected from different parasitic sites on each animal. Ticks from each animal were placed in an EP tube with breathable film to cover the punched holes in the top of the tube and prevent ticks escaping. Tick sample collection information is listed in Table 1.

Five milliliters of jugular blood was obtained from each animal by veterinarians, placed in an EDTA tube, and stored at −80 °C. A total of 195 domesticated animal blood samples were collected, of which 77.95% (152/195) were from goats, 13.33% (26/195) were from cattle, and 8.72% (17/195) were from dogs.

### 2.3. Nucleic Acid Extraction

Ticks were soaked in 75% ethanol and then in sterilized ultrapure water for 5 min each, washing three times to remove residual ethanol. Each group of ticks was flash-frozen in liquid nitrogen for 5 min in a 2 mL centrifuge tube; 200 μL of sterilized ultrapure water, 200 μL of proteinase K, and sterilized grinding beads were added; and the sample was ground using a cryogenic grinder (parameters: 25 Hz, 5–10 min) until completely lysed. To each tube was added 200 μL of anhydrous ethanol, vortexing for 30 s and centrifuging at 12,000× *g* for 5 min. The supernatant was used to extract nucleic acids with a DNA extraction kit (Beijing Tsingke Biotech Co., Ltd., Beijing, China) or an RNA extraction kit (Servicebio, Wuhan, China).

### 2.4. Tick Identification and Grouping

Ticks were classified according to morphology and representative ticks of each stage were confirmed with PCR amplification and DNA sequencing of the mitochondrial 16S RNA gene [21,26,27,28]. Ticks were grouped for RNA extraction according to their life stages and feeding status. The pooling strategy was designed based on the biological characteristics and feeding status of the ticks, aiming to balance detection sensitivity with practical feasibility. For feeding ticks, we used smaller pool sizes (1 engorged tick or 2–3 partially fed ticks), as because of their large body size, using individual or small pools for these specimens may reduce the risk of false negatives caused by dilution effects. For unfed ticks, we employed larger pool sizes (10–15 females, 15–20 males, or 30–40 larvae/nymphs) due to the small size of their bodies.

### 2.5. PCR and RT-PCR

The tick mitochondrial 16S rRNA gene was amplified using the PCR primers in Table 1, following a previously described protocol [21]. All PCR primers used in this study were synthesized by SinoBioscience (Shanghai, China). Ticks and animal blood RNA were reverse transcribed using the NovoScript^®^ Plus All-in-One 1st Strand cDNA Synthesis SuperMix (gDNA Purge) (Proxima Protein Technology, Suzhou, Jiangsu, China). Tick and animal cDNA were amplified with nested PCR for SFTSV L, M, and S segments, respectively. The PCR protocol was as follows: initial denaturation at 95 °C for 3 min; denaturation at 95 °C for 30 s; annealing for 30 s, with the annealing temperature for each pair of primers in Table 1; extension at 72 °C for 40 s, repeated for 35 cycles; and final extension at 72 °C for 10 min. The PCR primers are listed in Table 2.

The PCR product was separated with agarose gel electrophoresis, purified with a DNA gel extraction kit (Beijing Qikeweitech Co., Ltd., Beijing, China), cloned into the pMD 19-T vector (Takara Corporation), and transformed into DH5α competent cells. Positive clones were sequenced bidirectionally (Shenggong Biotechnology, Shanghai, China). The DNA sequences were assembled with the SeqMan Pro program in DNASTAR (7.0) software by removing the primer sequences, and they were compared with published sequences using BLAST (https://blast.ncbi.nlm.nih.gov/Blast.cgi) accessed on 14 August 2025. Selected sequences were analyzed with MEGA 12.0 software for phylogenetic analysis and phylogenetic trees were constructed using the maximum likelihood method based on the Kimura 2-parameter model.

## 3. Results

### 3.1. Tick Species in Suizhou City

We collected 2437 ticks, with the majority obtained from goats (72.7%, 1772/2437). *Haemaphysalis longicornis* accounted for 99.47% (2425/2437), *Rhipicephalus microplus* accounted for 0.45% (11/2437), and *H. flava* and *Ixodes sinensis* accounted for only 0.04% (1/2437), respectively.

### 3.2. Prevalence of SFTSV in Ticks and Animals from Suizhou City

The PCR results showed that one goat blood was positive for SFTSV S segment, four *H. longicornis* tick samples from goats were positive with the M segment, and one of them was also positive for the L segments. The positivity rate of SFTSV in goats was 0.66% (1/152), while the minimum infection rate of ticks (number of positive tick pools/total number of ticks) was 0.17% (4/2424) (Table 3). SFTSV was not detected in *R. microplus*, *Ixodes sinensis*, *Haemaphysalis flava*, cattle blood samples, or dog blood samples.

### 3.3. Phylogenetic Analysis of SFTSV from Ticks and Goats in Suizhou City

**Figure 2:**
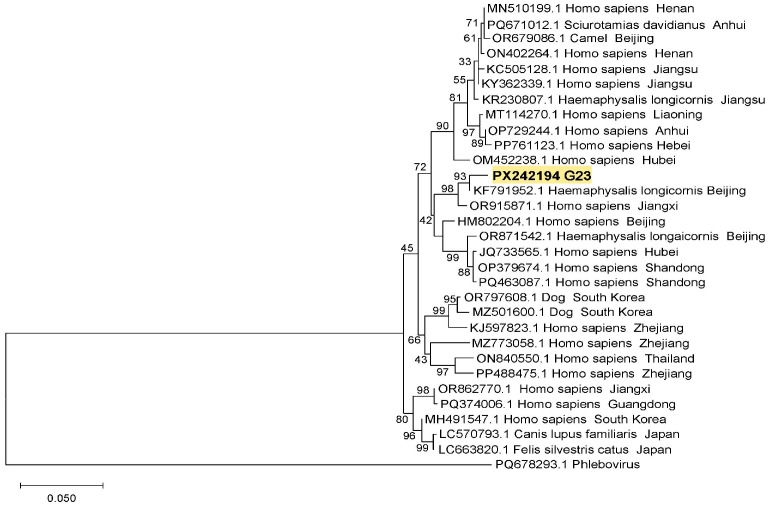
Phylogenetic tree of SFTSV S segment. G23 is the S segment sequence of SFTSV obtained from a goat blood sample in this study.

**Figure 3:**
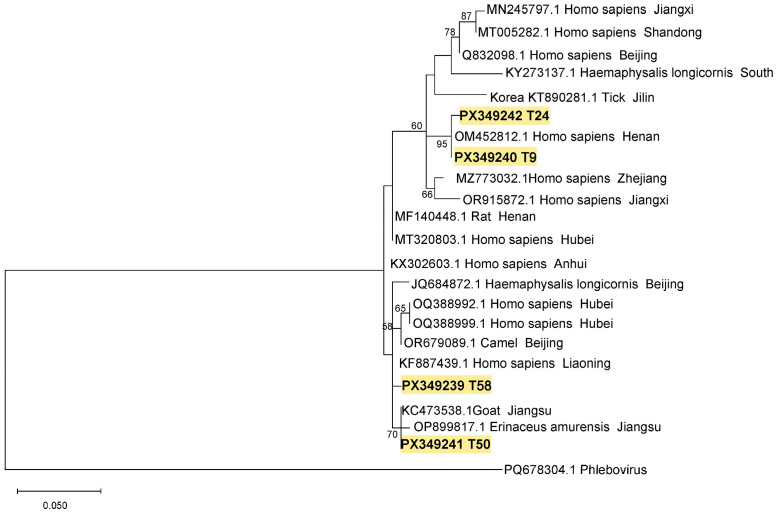
Phylogenetic tree of SFTSV M segment. Note: T9, T24, T50, and T58 are SFTSV M segment sequences obtained from ticks in this study.

The SFTSV sequences from ticks and goats were deposited in the GenBank with accession numbers of PX242194-PX242195 and PX349239-PX349242. DNA sequence analysis indicated that the S segment gene sequence (G23, PX242194) from one goat in this study was closely related (98.93% homology) to the sequence (KF791952.1) from a *H. longicornis* tick from Beijing, next to the sequence (OR915871.1) from a patient in Jiangsu Province with a nucleotide homology of 97.69% (Figure 2). In the phylogenetic tree, the SFTSV M segment sequences of T9 (PX349240) and T24 (PX349242) from ticks were 99.01% homology and were located on the same branch with an M segment (OM451812.1) from a patient in Henan Province, the M segments (MZ773032.1, OR915872.1) from patients in Zhejiang and Jiangxi provinces (Figure 3). The homology between gene sequences T58 (PX349239) and T50 (PX349241) was 98.51%, and they were closely related to the SFTSV M segments from a goat and a hedgehog in Jiangsu Province (KC473538.1, OP899817.1) (Figure 3).

**Figure 4:**
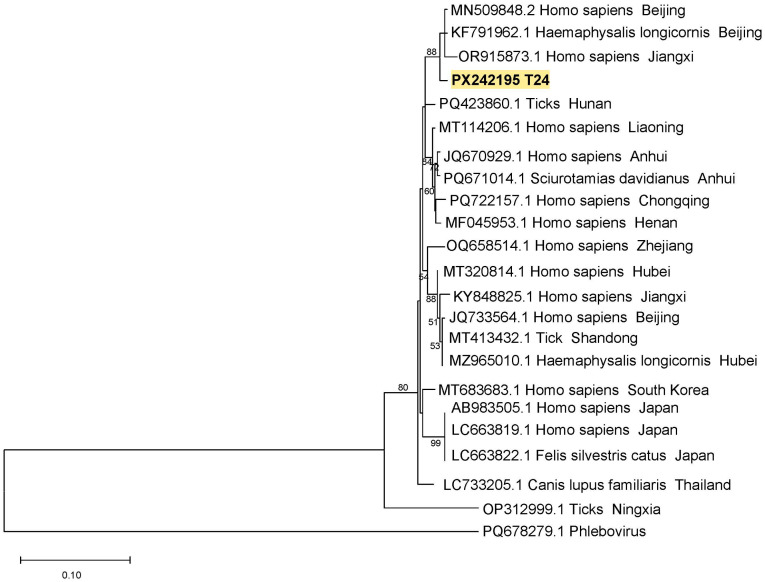
Phylogenetic tree of SFTSV L. Note: T24 refers to the SFTSV L segment sequence obtained from a tick in this study.

One tick (T24) was positive for SFTSV, with both the M segment and L segment. The L segment sequence (PX242195) was closely related to SFTSV strains from a patient and a *H. longicornis* tick from Beijing (MN509848.2 and OR915873.1) (Figure 4), with nucleotide homologies of 99.10% and 99.10%, respectively.

## 4. Discussion

Studies in eastern China and South Korea have proven that *H. longicornis* is the primary vector of SFTSV [21,22,23,24]; however, the vectors of SFTSV in central and western China and Southeast Asia are largely uninvestigated. In this study, we collected questing and feeding ticks in grasslands and domesticated animals in Suizhou City, an SFTSV endemic area in Hubei Province in central China. Four types of ticks were collected, including *H. longiconis*, *H. flava*, *R. microplus*, and *Ixodes sinensis*, with *H. longiconis* as the major tick species (99.47%). Most feeding ticks (96.20%, 1772/1842) were obtained from goats. We investigated the prevalence of SFTSV in ticks and livestock collected in Suizhou City. The infection rate of SFTSV in ticks (0.17%) from Suizhou City was consistent with previous studies from China and South Korea [21,23]. A study from an unspecified location in Hubei Province indicated that *H. longicornis* was negative for SFTSV [30]. Another study in Hubei found that the SFTSV infection rate of *Haemaphysalis longicornis* was 0.07% (3/4595) [25]. A study in Shandong Province showed that the infection rate of SFTSV in *H. longicornis* was 0.2% (8/3300) [21]. SFTSV has been found in several ticks species, including *H. longicornis*, *H. flava*, *H. concinna*, *Rhipicephalus microplus*, and *R. sanguinensis* [1,21,31,32,33,34]. Among these tick species, *H. longicornis* and *H. flava* have been demonstrated to be a reservoir and vector of SFTSV [21,32]. We did not found SFTSV in *H. flava*, *R. microplus*, or *Ixodes sinensis*, possibly due to small sample size of these tick species.

Our study demonstrated that *H. longiconis* is the primary tick species in central China, which is consistent with previous studies performed in East China and South Korea [21,23,24]. A large scale tick collection (27,029 ticks) from April to October in 2013, in South Korea, showed that *H. longiconis* was the primary tick species (64.76%) with *H. flava* second (29.22%) [35]. A year round study of ticks in South Korea demonstrated that among the 10,343 ticks collected from wild animals, *H. longicornis* constituted the majority (65.5%), followed by *H. flava* (33.8%) [23]. The prevalence of SFTSV-positive *H. longicornis* ticks peaked during the summer months and *H. flava* exhibited a higher prevalence during the winter season [23]. These studies indicated that both *H. longicornis* and *H. flava* are reservoirs of SFTSV in East Asia, but *H. longicornis* may be the more important vector for transmission of SFTSV because its peak activity aligns with agricultural planting times. *Haemaphysalis flava* has been reported on migratory birds [36,37], indicating *H. flava* may play a role in long distance transmission of SFTSV. *Haemaphysalis longicornis* has bisexual and parthenogenetic populations, and both populations are effective in transmission of SFTSV [21,38]. *Haemaphysalis longicornis* not only exists in East Asia, but has also spread to Australia, New Zealand, and the United States [39,40,41]. Metagenome sequence analysis of *Haemaphysalis longicornis* from China showed the tick harbored 48 virus species, with 22 novel viruses [42,43,44]. *Haemaphysalis longicornis* poses a risk of pathogen spillover due to its broad host range and ability to reproduce parthenogenetically [45].

A limitation of this study is that it focused only on endemic areas, without including non-endemic areas, making it difficult to infer regional risks or ecological patterns.

## 5. Conclusions

We found SFTSV in *H. longicornis* ticks and goats from Suizhou City, Hubei Provence, China. In SFTSV-endemic areas, *H. longicornis* tick is the primary tick species and goats are the main animal hosts for the *H. longicornis* tick, suggesting control of ticks on goats may play an important role in preventing SFTSV infection.

## Figures and Tables

**Table 1:** Tick sample collection information.

Source of Ticks		Adult Female Ticks	Adult Male Ticks	Nymphs	Larvae	Total
Free-living tick		203	56	305	31	595
Parasitic ticks	Goats	1248	328	183	13	1772
	Dogs	42	6	0	0	48
	Cattle	7	8	7	0	22
Total		1500	398	495	44	2437

**Table 2:** Primer information and PCR annealing temperature.

Tick and SFTSV	Primer	Primer Sequences	TEM (°C)	Size (bp)	Reference
Tick mitochondrial	F	AGTATTTTGACTATACAAAGGTATTG	55	402	
16S RNA	R	GTAGGATTTTAAAAGTTGAACAAACTT			
SFTSV	Out-F	CAGCCAGTTTACCCGAACAT	57	679	[20]
S segment	Out-R	GAAAGACGCAAAGGAGT			
	In-F	TGGCTCCGCGCATCTTCACA	57	560	
	In-R	TGGCTCCGCGCATCTTCACA			
SFTSV	Out-F	GRCATCTGARGCCAARTGYA	50	335	This study
M segment	Out-R	RACRTGKATWGCTRYTTTYCC			
	In-F	AARCCYGGRGAAGTYGTWGT	50	278	
	In-R	CRACYARYGAYCCWGARTGGA			
SFTSV	Out-F	AATGATGCCAAGAAGTGGAAT	52	855	[29]
L segment	Out-R	ATGTAAGCATAGTCCTAGAAGC			
	In-F	CCACAGATTCATTTGGGCT	55	367	
	In-R	ATCATGATCGCTGAGTCGTC			

Note: TEM, annealing temperature.

**Table 3:** Prevalence of SFTSV in samples collected in Suizhou, Hubei, China.

Host	Infection Rate
*Haemaphysalis longicornis*	0.17% (4/2424)
Goats’ blood samples	0.66% (1/152)

## Data Availability

The original contributions presented in the study are included in the article, further in-queries can be directed to the corresponding authors.
